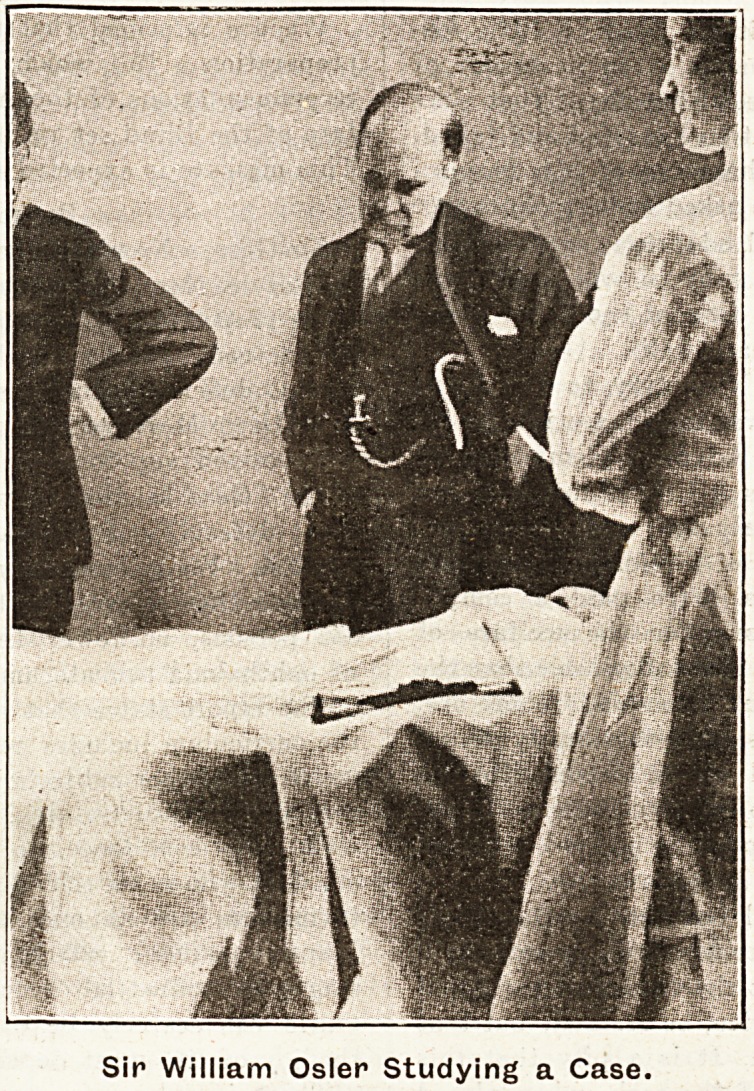# Sir William Osler and Oxford

**Published:** 1920-01-17

**Authors:** 


### SIR WILLIAM OSLER AND OXFORD.

### A Great Living, Life-Giving Personality.

THE power of any life lies in its expectancy. What do you hope for, what do you expect? And the answer to these questions is the measure of the degree in which any man is alive and taking his place in the world.

There can be little doubt that in Oxford Sir William Osler's untiring energy and enthusiasm, the place he filled, was the result of a personality which took its direction from unbounded hope. His was that splendid buoyancy of spirit which was content to know that there is always a going forward in the world to which he could link his labour without spending it in vain; content if a man is only shown his work, content that, whatever in this world proves disappointing, there is one hope that maketh not ashamed—the love of God and the love of man.

*Photo.]* *[Elliot and Fry.*

THE LATE SIR WILLIAM OSLER, M.D., F.R.S.

Throughout his life right on to the end he retained, like all great men, a vein of boyishness which charmed and inspired. His was that sunny, genial spirit, unsoured by suspicion, unburdened by care, a spirit not in the least ashamed to enjoy everything heartily, to reverence humbly, to admire unreservedly, to love and trust with a whole heart. In a word, the spirit of a real boy, which retained its bloom and grace and fragrance long after the cool morning hours had changed into the glare and dust and heat of midday.

Sir William made every man do his best, and tried to bring out that he had within him, simply by this wonderful quality which none failed to perceive within him, and which touched every department of his activities here.

As Regius Professor of Medicine he was a member of Christ Church, and although it was in the middle of the Christmas vacation when the funeral service was held at the Cathedral, there was not a member of the governing body—not a single member of the University who could by any means get back, who did not attend to pay the last homage to the man who had made us all feel he was our "brother," and to whose memory there were no words that could be found to say what each felt, and we could but clasp each other's hand.

On the governing body of Christ Church he brought a wide knowledge and experience, and to its library he gave a large and valuable collection of books. A great collector of books himself, he loved to spend spare hours in the College libraries, among the presses, and there were few early books on medicine which he did not know something, if not all, about, and, like all his knowledge, it was at hand and came out instantly in all sorts of ways.

He was curator of the Bodleian Library, and took enormous pains and interest in the work of

**Sir William Osler and Oxford**—(*continued*)

directing the policy and administration of this great library. He had views on the storing of books, he knew the library systems in all parts of the world, had seen methods of cataloguing in a hundred different libraries, he knew of "book worms" and "moulds," the enemies of books, and laid all his knowledge at the disposal of the librarian, and did it, as he did all his work, with an ease and a grace which made it quite impossible to feel annoyed even if he criticised some slackness or some backward tendency rather unmercifully.

He could generally get things done, even in so-called unprogressive academic circles. To have a portion of the gallery of the Radcliffe Camera fenced off, and to have brought a very large collection of books connected with the History of Science and there to set down two learned people to work at this subject —in a workshop as it were with every tool to hand—was a great achievement, and one which has already borne fruit in the publication of early documents connected with the subject. When first he got this "study" together, if one met Sir William anywhere near the Camera, he would at once carry one off to see it, to be introduced to the workers there and to direct attention to a priceless MSS. just unearthed anew, and being transcribed. However busy one was, no refusal was allowed. Sir William had you under his spell.

Being a delegate of the University Press, it was but natural that he could find fresh uses for it, for the subject of medicine began to appear in its catalogues more frequently, and learned medical periodicals came to be published here. He thought the Bodleian should make its priceless stores better known to the outside public, and for many years he paid the deficit of the publication of a quarterly journal setting forth most of the recent additions to the library, articles on the history and methods of administration or its *personnel*, and certainly made a brochure which was of very great interest and value. It told the University a great deal which its apathy would otherwise pass over. Sir William ever loved to encourage and reward sound work of any sort, and he will be missed at the Bodleian and the Press as much as anywhere.

The Regius Professor of Medicine, who is not rewarded handsomely by this University, possesses almost as his sole emolument a house as warden at the beautiful and ancient Alms House at Ewelme. One of his first activities was to restore and set this house in order and investigate its antiquities and its history.

Unlike his predecessors (Sir Henry Acland and Sir Burdon Sanderson) in this chair, he was a clinician intensely interested in hospitals and their work. He took a class round the Radcliffe Infirmary regularly once, and often twice, a week, and doctors as well as the beginners in medicine walked round with him. It was an experience in many ways. Sisters, nurses, everyone knew Sir William's "days," and always felt reinvigorated by his visits.

The treasurer of this hospital was once talking to a sister in her ward when Sir William came in with his class. Sister was just leaving to get married. As he passed into the ward he said to the treasurer, "Are you inducing cardiac dissociation between sister and her young man? I hope so—she must not be allowed to leave me." That was all; he then passed on to cheer someone up—a child, a nervous patient—or to unravel a knotty tangle in a diagnosis of a house physician.

Nothing was more wonderful than the way in which he would call for the notes—the temperature charts of a patient he was seeing for the first time lay all out on the bed—and saying without a moment's hesitation, "The last time I saw a temperature like this was with my dear old friend James William Jones of Philadelphia. How he had puzzled over it, and this makes only the 103rd example I know of." Then came a wonderful array of knowledge, perhaps the outlines of a new treatment to be tried, and the class passed on to admire the wonderful insight and instant touch of a great man absolutely sure of himself.

He was always at the call of any of the medical staff of the hospital, and would often look in to ask the house men if there was anything he could do for them. Sometimes it was merely his intense sympathy which alone was required. During the war a small Belgian child was brought in with pneumonia, and the exiled mother, who could but imperfectly understand English, was stricken with grief at this illness of her only child.

Sir William came into the hospital, and matron took him to see the mother to comfort her. He examined the wee mite, stroked her hair, and tenderly treated her, as he ever did children, whom he loved to have around him at home. He left, and the mother, not knowing who he was, was told that he was "the doctor who, if the King had been taken ill in Oxford, would have been sent for to see him." She was overcome with gratitude, and dried up her tears, saying in her broken English—"I am so glad"—"I am so thankful." "It is so good." "He knew."

Sir William was at his best at meetings of doctors, and at any meeting he could ever say what he wanted with such point that none could ever forget it. At a meeting of Oxford medical graduates he begged the doctors not to forget their Latin. "You put *R* at the top of your prescriptions, because you don't know the imperative of the Latin verb "to take." You call it an appeal to Jove to help you, but it is sheer ignorance that prevents you writing it out in full. And as for *syr.* or *co.* you stop there because none of you have the least idea what the genitive of *syrupus* is, or the rest of the word beginning with *co.*"

It was at the same meeting that he had been earlier in the day to see a collection of paintings of a "post-impressionist school," and he was greatly disgusted, telling the doctors that it was "pre-adamite," not "post-impressionist," and that there was not a single *normal healthy face* in the whole collection, but as an example of skin diseases and evidence of medical defects they might visit the exhibition for their information and use, and the

**Sir William Osler and Oxford**—(*continued*).

whole better had go to a museum of morbid anatomy.

At a meeting of the City Welfare Association in Oxford he was always welcome (it was his usual practice to help the City as much as the University). He criticised the housing of Oxford, the slums, the slow way the City fathers worked at a scheme for tuberculosis, or anything that struck him as ineffective or lacking in go, but always by a little touch which could not help go home—*e.g.*, "Afraid of being near a tuberculosis dispensary! Why I would put my house next door if I were building one, because it would be the only place I know where they could keep the bugs chained up."

On one occasion he was in the chair of an Infant Welfare Association, and had heard in the report that more *perambulators* were required. In summing up at the end he said this deficit could easily be remedied that afternoon as he saw five bachelors in front of him, and he proposed to tax them there and then a pound apiece. And he did to make up the £5 necessary.

**Sir William Osler Studying a Case.**

There is no department of life in Oxford where the loss of Sir William will not be felt at once. Although he had been ill for three months, none outside his immediate circle knew that the end was so near, and when it came the whole of Oxford mourned and went still and quiet.

The service in the cathedral on New Year's Day was a wonderful tribute to him, not because so many great and distinguished men were present, but because everyone seemed to feel what had happened, and everyone came away as one who mourned a man who had been "a brother" to him, one who had given him better things to do, helped him along a higher path of duty and service, and given him inspiration and courage for it all.

Probably the best and fullest account of Osler's life-work will be found in the *Bulletin* of the Johns Hopkins Hospital, Baltimore, for July 1919.

## Figures and Tables

**Figure f1:**
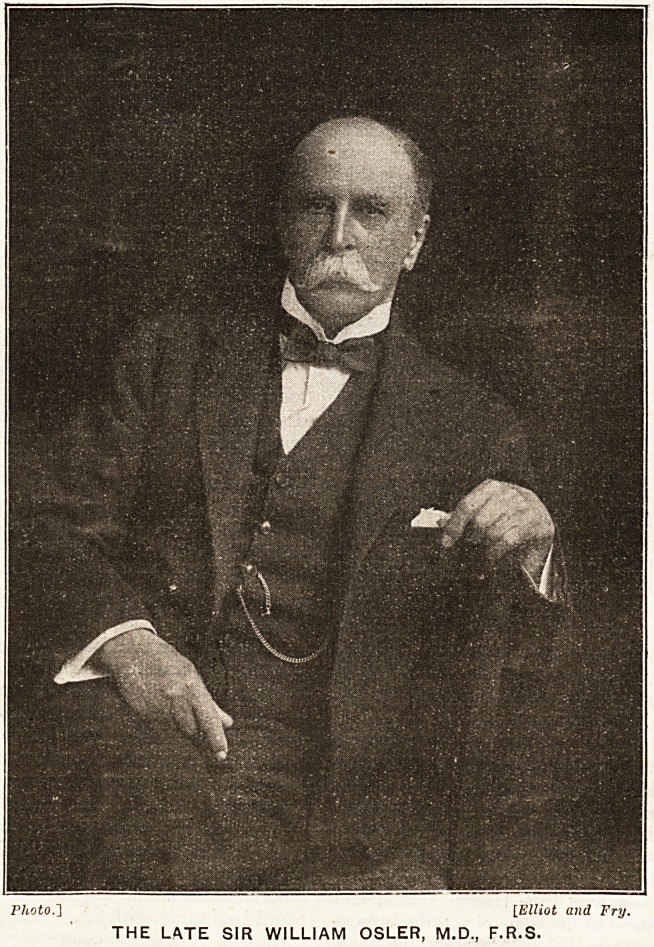


**Figure f2:**